# Phase-locking of bursting neuronal firing to dominant LFP frequency components

**DOI:** 10.1016/j.biosystems.2015.08.004

**Published:** 2015-10

**Authors:** Maria Constantinou, Daniel H. Elijah, Daniel Squirrell, John Gigg, Marcelo A. Montemurro

**Affiliations:** Faculty of Life Sciences, The University of Manchester, Manchester M13 9PT, UK

**Keywords:** Bursting, Local field potential, Phase-locking, Subiculum, Neural coding

## Abstract

Neuronal firing in the hippocampal formation relative to the phase of local field potentials (LFP) has a key role in memory processing and spatial navigation. Firing can be in either tonic or burst mode. Although bursting neurons are common in the hippocampal formation, the characteristics of their locking to LFP phase are not completely understood. We investigated phase-locking properties of bursting neurons using simulations generated by a dual compartmental model of a pyramidal neuron adapted to match the bursting activity in the subiculum of a rat. The model was driven with stochastic input signals containing a power spectral profile consistent with physiologically relevant frequencies observed in LFP. The single spikes and spike bursts fired by the model were locked to a preferred phase of the predominant frequency band where there was a peak in the power of the driving signal. Moreover, the preferred phase of locking shifted with increasing burst size, providing evidence that LFP phase can be encoded by burst size. We also provide initial support for the model results by analysing example data of spontaneous LFP and spiking activity recorded from the subiculum of a single urethane-anaesthetised rat. Subicular neurons fired single spikes, two-spike bursts and larger bursts that locked to a preferred phase of either dominant slow oscillations or theta rhythms within the LFP, according to the model prediction. Both power-modulated phase-locking and gradual shift in the preferred phase of locking as a function of burst size suggest that neurons can use bursts to encode timing information contained in LFP phase into a spike-count code.

## 1 Introduction

Local field potentials (LFP) are fluctuating extracellular electrical signals that result from the sum of currents across all excitable membranes within a local volume (Logothetis, 2003, Buzsaki et al., 2012). A major contributor to the LFP is the combined synaptic activity of neuronal populations (Einevoll et al., 2007, Pettersen et al., 2008). Neuronal firing relative to the phase of ongoing LFP oscillations in the hippocampal formation has been linked with spatial navigation (O’Keefe and Recce, 1993, Skaggs et al., 1996) and memory processing (Lisman and Idiart, 1995). Moreover, evidence from the monkey sensory cortices suggests that more information about stimuli can be transmitted if the LFP phase at which spikes are fired is taken into account (Montemurro et al., 2008, Kayser et al., 2009). The phase of LFP oscillations has been proposed to be involved in keeping timing information for neural communication (Fell and Axmacher, 2011). Thus, locking of neuronal firing during a preferred phase range can be a mechanism of transmitting information for cognitive processing. Although pyramidal neurons in the hippocampal formation are known to lock their firing to LFP phase, the conditions of this phase-locking are not completely understood.

Two factors that may affect the locking properties of pyramidal neurons are the frequency composition of the LFP and the dynamics of individual neurons. The former is relevant to the hippocampal formation since this area is characterised by well-defined oscillatory states that correlate with cognitive function. Regarding neuronal dynamics, pyramidal neurons can fire in either tonic or bursting modes. The existence of bursting neurons in the hippocampal formation has been long documented (Ranck, 1973). Bursting activity is an important mechanism for neural communication because bursts consisting of different spike counts can provide more basic symbols in the neural code (Kepecs and Lisman, 2003, Samengo et al., 2013). Modelling studies have provided some evidence that bursting pyramidal neurons can lock to different phases of the LFP depending on the burst spike-count (Samengo and Montemurro, 2010), but this has not been tested in specific models based on experimental data. We address this by studying phase-locking of bursting activity in the subiculum which is known to contain intrinsically bursting neurons (Sharp and Green, 1994, Gigg et al., 2000). The subiculum is the major output structure of the hippocampus (for reviews on the subiculum see O’Mara et al., 2001, Gigg, 2006). Similarly to the hippocampus, neurons in the subiculum encode spatial information (Kim et al., 2012) as for example the boundary vector cells which are neurons that fire when a rat encounters boundaries in space (Lever et al., 2009).

We used a neuron model and example *in-vivo* data from the subiculum of a single rat to investigate how bursting neuronal dynamics and LFP frequency components affect phase-locking. The model predicted that bursting neurons locked their firing to a preferred phase of dominant rhythms irrespective of the frequency of these rhythms and phase preference shifted with increasing spike count. We show that subicular bursting neurons locked their firing to a preferred phase of dominant slow oscillations or theta rhythms within the LFP and the preferred phase of locking to dominant slow oscillations changed depending on the spike count according to model predictions. These results suggest a mechanism of encoding timing information in burst spike-count.

## 2 Materials and methods

### 2.1 Bursting neuron model

**Fig. 1:**
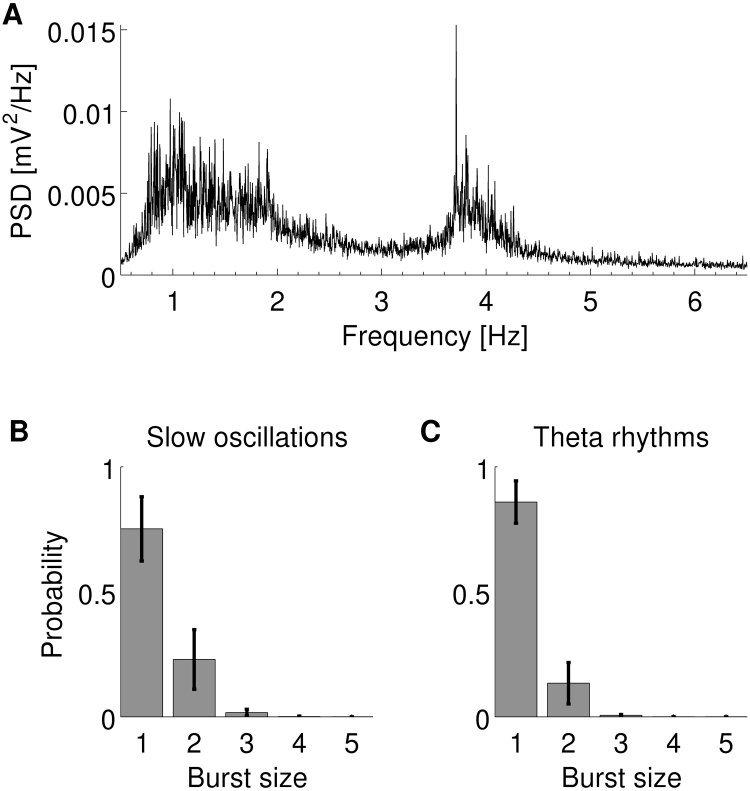
(A) Average power-frequency spectrum of LFP recordings in the rat subiculum under urethane anaesthesia. During the 1-h recording, there were two spectral peaks: a wide peak at 1–2 Hz and a sharper peak at about 4 Hz. (B and C) Average probability of a bursting neuron in the subiculum of a urethane-anaesthetised rat firing an *n*-spike burst when slow oscillations (B) or theta rhythms (C) were dominant in the LFP. The errorbars indicate standard deviation.

**Fig. 2:**
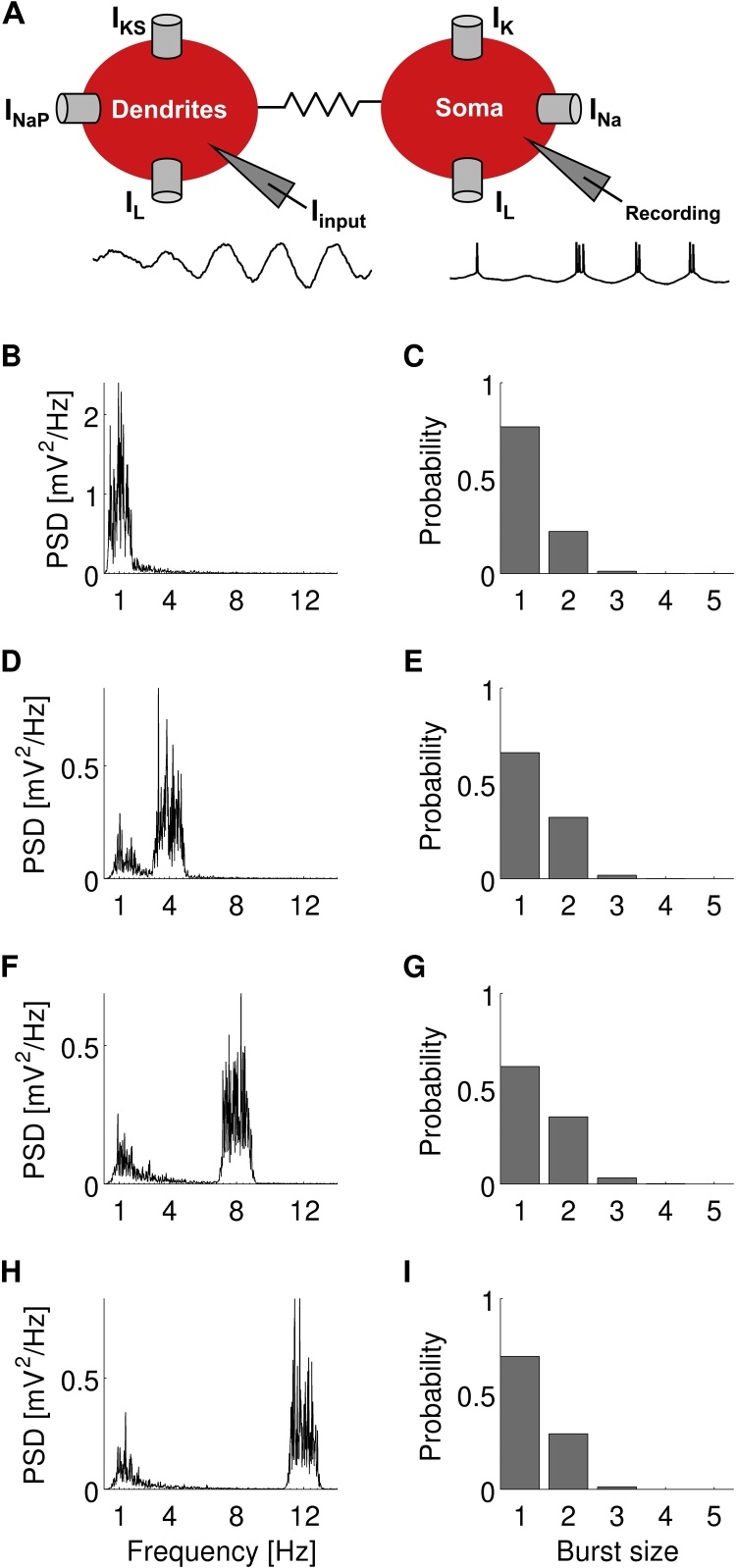
(A) Diagram of the two-compartment model of a bursting pyramidal neuron showing the ionic currents. The input signal *I*_*input*_ is injected into the dendritic compartment and bursting activity is recorded from the somatic compartment. (B, D, F and H) Power-frequency spectra of input signals to the model. The input signal consists of background coloured noise and a power spectral peak at either 1 Hz (B), 4 Hz (D), 8 Hz (F) or 12 Hz (H). (C, E, G and I) Probability of the model firing an *n*-spike burst when the input signal comprised the frequencies depicted in the plots at the left.

A two-compartment (dendrites and soma) conductance-based model of a bursting pyramidal neuron was used for all simulations (Fig. 2A). This model is a reduction of a 19-compartment (Traub et al., 1991) to a two-compartment model of a CA3 hippocampal neuron (Pinsky and Rinzel, 1994) which was simplified by Kepecs and Wang (2000) to include the minimal ionic conductances required to generate bursting. This model has been used to investigate the properties of bursting in response to different stimuli in previous studies (Kepecs et al., 2002, Kepecs and Lisman, 2003, Samengo and Montemurro, 2010). For the purpose of this study, the model was fitted to match its responses to realistic burst firing in the rat subiculum. To achieve this, we used the equations and parameters as described in Samengo and Montemurro (2010) and adjusted four parameters (*g*_*K*_, *g*_*NaP*_, *g*_*KS*_ and *C*_*m*_) so that the probability of firing bursts of size *n*, where *n* is the number of spikes in a burst and *n* = 1 for single spikes, is similar to the firing probability of subicular neurons (Fig. 1, Fig. 2).

An input current *I*(*t*) injected into the dendritic compartment produced bursting activity in the somatic compartment according to:

(1) $$C_{m}\frac{{\mathrm{dV}}_{d}}{\mathrm{dt}}=-I_{L}-I_{\mathrm{KS}}-I_{\mathrm{NaP}}-g_{c}\frac{V_{d}-V_{s}}{1-p}+I(t)$$

(2) $$C_{m}\frac{{\mathrm{dV}}_{s}}{\mathrm{dt}}=-I_{L}-I_{K}-I_{\mathrm{Na}}-g_{c}\frac{V_{s}-V_{d}}{p}$$

The relative area between the two compartments was *p* = 0.15 and the coupling conductance was *g*_*c*_ = 1 mS/cm^2^. The somatic compartment included a Na current: $I_{\mathrm{Na}}=g_{\mathrm{Na}}m_{\infty }^{3}h(V_{s}-E_{\mathrm{Na}})$, where *m*_∞_ = *α*_*m*_/(*α*_*m*_ + *β*_*m*_), *α*_*m*_ = −0.1(*V*_*s*_ + 31)/(exp(−0.1(*V*_*s*_ + 31)) − 1), *β*_*m*_ = 4 exp(− (*V*_*s*_ + 56)/18), *α*_*h*_ = 0.07 exp(− (*V*_*s*_ + 47)/20) and *β*_*h*_ = 1/exp(−0.1(*V*_*s*_ + 17)) + 1); and a K current: *I*_*K*_ = *g*_*K*_*n*^4^(*V*_*s*_ − *E*_*K*_), where *α*_*n*_ = −0.01(*V*_*s*_ + 34)/(exp(−0.1(*V*_*s*_ + 34)) − 1) and *β*_*n*_ = 0.125 exp(− (*V*_*s*_ + 44)/80). The dendritic compartment included a persistent Na current: *I*_*NaP*_ = *g*_*NaP*_*r*_∞_^3^(*V*_*d*_ − *E*_*Na*_), where *r*_∞_ = 1/(exp(− (*V*_*d*_ + 57.7)/7.7) + 1); and a slow K current: *I*_*KS*_ = *g*_*KS*_*q*(*V*_*d*_ − *E*_*K*_), where *q*_∞_ = 1/(exp(− (*V*_*d*_ + 35)/6.5) + 1), $\tau_{q}=\tau_{q_{0}}/(\exp(-(V_{d}+55)/30)+\exp((V_{d}+55)/30))$ and $\tau_{q_{0}}=200$. The leak currents were described by *I*_*L*_ = *g*_*L*_(*V* − *E*_*L*_), where *V* is *V*_*d*_ or *V*_*s*_. Each gating variable *x* followed the kinetics equation: *dx*/*dt* = *ϕ*_*x*_(*α*_*x*_(1 − *x*) − *xβ*_*x*_) = *ϕ*_*x*_(*x*_∞_ − *x*)/*τ*_*x*_. The maximum conductances (in mS/cm^2^) were *g*_*Na*_ = 45, *g*_*K*_ = 15, *g*_*L*_ = 0.18, *g*_*NaP*_ = 0.08, *g*_*KS*_ = 0.7 and the reversal potentials (in mV) were *E*_*Na*_ = 55, *E*_*K*_ = −90, *E*_*L*_ = −65. Membrane capacitance was *C*_*m*_ = 0.6 μF/cm^2^. The temperature scaling factors were *ϕ*_*h*_ = *ϕ*_*n*_ = 3.33 and *ϕ*_*q*_ = 1. The model was integrated with the 4th order Runge–Kutta method with a time step of 0.01 ms.

### 2.2 Input to the model

LFP are broadband signals containing power spectral peaks within frequency bands which are usually associated with different behavioural states. The input to the model was a time-varying signal which simulated physiologically relevant rhythms present in LFP. To obtain this input, a signal containing one peak at a selected frequency in the power-frequency spectrum was added to a background coloured-noise signal. The background signal simulated low-power oscillations and temporal correlations present in LFP. To generate the background signal, a white-noise process was convolved with an exponential kernel and then high-pass filtered with a 3rd order Butterworth filter with a cut-off frequency of 1 Hz to remove low frequency components. To create the signal with a peak in power at a given frequency, a white-noise process was narrowband filtered with a Kaiser filter (width of band was 1 Hz) so that the signal contained only a sharp peak centred at either 1, 4, 8 or 12 Hz in the power-frequency spectrum. The background coloured-noise and frequency peak signals were scaled to have standard deviation of 0.02 and 0.03, respectively, and then added together. The resulting signal was scaled again to have mean *μ* = 0 and standard deviation *σ* = 1.2 for the 1 Hz peak and *σ* = 0.8 for the remaining three peaks. This difference in standard deviations was required to reflect that slow oscillations have higher amplitude compared to higher frequency rhythms. Assuming that the LFP can be simulated by the sum of synaptic inputs to neurons (Mazzoni et al., 2008), the input was injected as current into the dendritic compartment of the model (Eq. 1).

### 2.3 *In vivo* electrophysiology

All experimental procedures were carried out in accordance with the Animals (Scientific Procedures) Act UK 1986. Ethical approval was provided by the University of Manchester Ethical Review Panel. *In vivo* electrophysiological recordings of LFP and spiking activity were obtained from an adult male Sprague Dawley rat (Charles River, UK: 332 g, group-housed in a pathogen-free environment with food and water available *ad libitum*, maintained on a 12-h light:dark cycle).

Initial anaesthesia was induced via i.p. injection of urethane (30%, w/v in 0.9% saline, 1.8 g/kg) and top-up doses of urethane (between 0.1 and 0.15 ml) were administrated at approximately 30-min intervals until areflexia was achieved. Body temperature was kept at 37 °C using a homeothermic heating pad. The rat was head-fixed in a stereotaxic frame and a 2-mm diameter craniotomy was carried out according to the Paxinos and Watson (2007) rat brain atlas for the subiculum (Bregma: −8.0 mm, ML: 3.5 mm). The dura was excised and a 4 × 8 multi-electrode array (A4×8-5-50-200-413, NeuroNexusTech, USA) was inserted at a 30 ° compound angle from the vertical axis to match the main dendritic axis of the subiculum. The electrode array was composed of four shanks, each containing eight 413-μm^2^ electrodes with 50 μm vertical and 200 μm horizontal spacing between electrodes/shanks, respectively. The array was attached to an electrode board and headstage (Plexon, USA) with fixed gain of 20× and an AC preamplifier providing a total gain of 2000× (Recorder64, Plexon, USA). The positions of the electrodes were verified from Nissl-stained brain sections (Fig. S1) by detecting small electrolytic lesions produced by applying a 30 μA current for 5 s (Townsend et al., 2002) at the end of the experiment.

Spontaneous LFP (2 kHz sampling rate, low-pass filtered up to 250 Hz) and spiking activity (40 kHz sampling rate, high-pass filtered above 300 Hz) were recorded simultaneously from the electrodes in subiculum for 1 h. Recordings were ground referenced to the stereotaxic frame. Spikes were detected online by manually setting a threshold for each electrode and stored as discrete shapes (1.3 ms duration) for offline spike sorting.

### 2.4 Data analysis

#### 2.4.1 Spike sorting

To identify spikes fired by individual neurons, the recordings of spike shapes were analysed using Offline Sorter V2.8.8 (Plexon Inc). Different spike shape parameters were clustered until units were distinguished from the ‘noise’ cluster and manually separated. The separation quality was assessed by visually inspecting the interspike interval (ISI) histogram for each unit to ensure there were no spikes within the 1 ms refractory period. Multiple detections of the same unit on adjacent electrodes were identified by plotting cross-correlograms of each unit versus every other unit and only the unit with the largest waveforms was kept for each duplicate.

#### 2.4.2 Spectral analysis and data segmentation

Spectral analysis was done using the Welch's periodogram method with 50% overlapping Hamming windows of length 112.5 s or 450 s for the input signal to the model or LFP signals, respectively. The 1-h LFP signals contained two spectral peaks: at slow oscillations and theta rhythms (Fig. 1A). To segment the LFP signals into epochs containing only one dominant rhythm, the power distribution over the frequency ranges 0.5–2.5 Hz for slow oscillations and 2.5–5.0 Hz for theta rhythms was estimated at every time point from the Fourier time-frequency decomposition over Hamming windows of 2.048 s with 50% overlap. The power over these frequency ranges was integrated to compute how much power as a percentage of the total was in each band. The dominant rhythm at a given time point was defined as the one which had at least 10% higher power than the other. That is, the fraction of total power within the dominant band was at least 0.1 greater than the fraction within any other frequency band. The 10% margin was sufficient to identify epochs of LFP with dominant rhythms in our data recorded under urethane anaesthesia as shown in the power spectra of the segmented data in Fig. S2.

#### 2.4.3 Spike segregation into bursts

The spike times recorded for each unit were separated into two datasets depending on whether spikes were fired when slow oscillations or theta rhythms were dominant. Units were classified as bursting if in the ISI histograms and autocorrelograms of spike times there was a sharp peak within 2–8 ms and this peak was larger than any other peak within 50 ms. To segregate spikes fired by subicular neurons into bursts, an ISI threshold of 8 ms was chosen because this time point was after the ISI histogram peak which indicated the time interval between spikes within bursts. A spike was considered as part of a burst if the spike occurred within 8 ms from the previous spike in the burst. If the interval between two spikes was greater than 8 ms, the spikes were considered as separate events. For segregating burst spikes fired by the model, an ISI threshold of 10 ms was used because the sharp peak in the ISI histograms and autocorrelograms occurred within 2–10 ms.

#### 2.4.4 Filtering and phase extraction

Both the LFP recordings from the rat subiculum and the input signals to the model were downsampled to 500 Hz. Filtering was carried out with a finite impulse response (FIR) digital filter with Kaiser window (sharp transition bandwidth: 1.0 Hz, stopband attenuation: 60 dB, passband ripple: 0.01 dB). The signals were filtered in narrow bands of 1 Hz with 25% overlap, apart from the first band which ranged from 0.1 Hz to 1 Hz. The centres of the narrow bands were at 0.55 Hz, 0.75 Hz and then increased in steps of 0.25 Hz up to 10.25 Hz or 14.25 Hz. Phase was extracted as the argument of the Hilbert transform of the filtered signals. A phase value of 0° corresponded to the peak of an oscillation. For all phase analyses, we used the phase of the filtered signals at the time of spike or burst onset.

#### 2.4.5 Phase-locking estimation

Phase-locking was estimated using histograms because this method captures both the strength of locking and the distribution of preferred phases. A waveform cycle from −180° to 180° was separated in either 125 bins of size 2.88° for the simulations or 25 bins of size 14.4° for the experimental data. The difference in the number of bins was because we used the model to simulate enough data to allow for finer binning than was allowed by the finite number of events fired by subicular neurons during the recording session. For the model, phase-locking histograms were constructed by calculating the probability of a spike or burst being fired within each phase bin of the narrowband-filtered input signal. For the experimental data, phase-locking of spikes and bursts was calculated relative to the LFP recorded at the same electrode where the spiking activity of the unit was recorded. Average phase-locking histograms were obtained by averaging the probabilities of firing spikes and bursts within each phase bin of the narrowband-filtered LFP across bursting units in epochs when slow oscillations or theta rhythms were dominant. To accommodate for differences in phase preference of individual units (examples in Figs. S3 and S4), the phase of 0° was set as the phase of mean maximal locking of single spikes and phase-locking of spikes and bursts fired by each unit was calculated relative to that phase. Mean and standard deviation of the phase-locking distributions were calculated using the circular statistics toolbox for Matlab (Berens, 2009).

## 3 Results

We investigated bursting activity in relation to LFP using a computational approach. We first present the experimental data which were used to match the firing statistics of the neuron model. We then present results of extensive simulations of the model where we explored the locking properties of spikes and bursts of different spike count. Finally, we provide an example from subicular bursting neurons illustrating that the patterns predicted by the model are also present *in vivo*.

### 3.1 Bursting neurons in subiculum

In order to match the firing statistics of the neuron model to realistic burst firing in the subiculum, we analysed 1-h multi-electrode recordings of simultaneous LFP and spikes from the subiculum of a urethane-anaesthetised rat. The power spectrum of the LFP contained a wide peak around 1–2 Hz and a sharp peak at about 4 Hz (Fig. 1A). The first frequency peak is often referred to as slow oscillations or delta rhythms and the latter as theta rhythms. These two states under anaesthesia are analogous to non-REM and REM sleep, respectively (Clement et al., 2008). Since different frequency bands might correspond to different cognitive processes, we analysed epochs of dominant slow oscillations and theta rhythms separately. Out of a total of 26 units identified in the rat subiculum, we identified 13 bursting units firing at a rate of 1.96 ± 1.00 events/s in epochs when slow oscillations were dominant in the LFP. Eleven of these units were also bursting with a firing rate of 3.83 ± 2.68 events/s when theta rhythms were dominant. All bursting units fired single spikes and bursts comprising two or more spikes at a decreasing probability (Fig. 1B and C). Bursts consisting of three or more spikes were rare so were grouped together for the following analyses.

### 3.2 Bursting neuron model

To explore the phase-locking properties of bursting neurons, we adapted a dual compartmental model of a bursting pyramidal neuron (Fig. 2A). The model was driven with an input comprising time-varying stochastic signals with a peak in the power-frequency spectrum in order to simulate similar frequencies occurring in LFP signals when there is only one dominant rhythm. The peaks were centred at 1 Hz (Fig. 2B), 4 Hz (Fig. 2D), 8 Hz (Fig. 2F) and 12 Hz (Fig. 2H). The peak at 1 Hz simulated dominant slow oscillations which are characteristic during sleep and anaesthesia. The peak at 4 Hz and 8 Hz simulated dominant low and high theta rhythms, respectively. Low theta rhythms are observed under urethane-anaesthesia and high theta rhythms are prevalent during awake exploratory behaviour. The peak at 12 Hz corresponded to dominant alpha rhythms which are higher than the frequencies usually found to be dominant in the LFP recorded from the rat hippocampal formation *in vivo*. The model fired *n*-spike bursts (Fig. 2C, E, G and I) in response to these four input signals with similar positively skewed probability distributions as the bursting units in the rat subiculum (Fig. 1B and C).

### 3.3 Spikes and bursts lock to phase of dominant rhythms

**Fig. 3:**
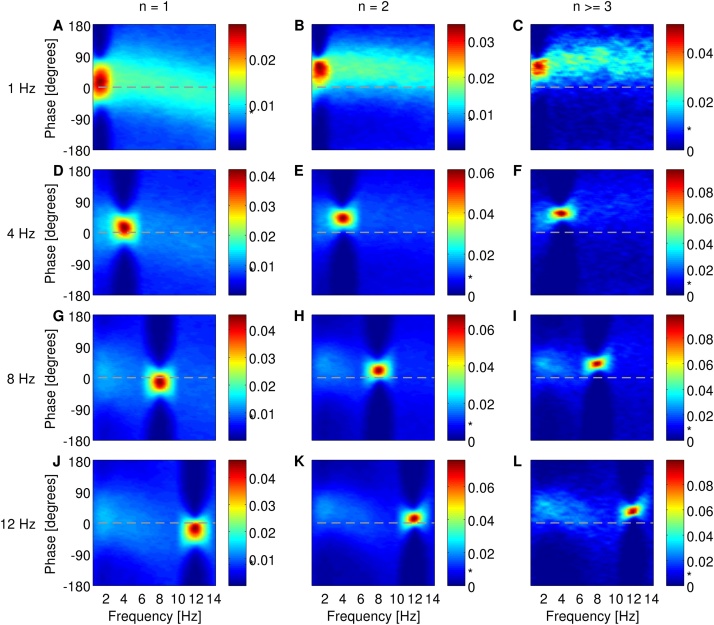
Phase-locking histograms of single spikes (A, D, G and J), two-spike bursts (B, E, H and K) and larger bursts (C, F, I and L) fired by the model when the input signal contained a frequency peak at 1 Hz (A–C), 4 Hz (D–F), 8 Hz (G–I) or 12 Hz (J–L). Phase of 0° corresponds to the peak of a waveform as calculated by the Hilbert transform. The colourbar shows the probability of locking to the phase of filtered signal at overlapping steps of 1 Hz. The asterisk (*) in the colourbar indicates chance probability which is equal to 1/125 or 0.008. (For interpretation of the references to colour in text, the reader is referred to the web version of the article.)

Is phase-locking of bursting neuronal firing to LFP rhythms an intrinsic property of bursting neurons regardless of the frequency of these rhythms or is locking restricted to specific frequency bands irrespective of their power? To address this, we used the model to simulate bursting activity in response to broadband signals with spectral peaks at different frequencies resembling LFP containing only one dominant rhythm. If neuronal activity is phase-modulated by oscillations within specific frequencies, then neurons should fire with a high probability at a preferred phase of these oscillations. Instead, if neuronal activity is independent of the phase of a specific rhythm, then the firing probability should have a flat distribution relative to the phase of this rhythm. The single spikes (*n* = 1), two-spike bursts (*n* = 2) and larger bursts (*n* ≥ 3) fired by the model were locked to a preferred phase of the dominant rhythm within the input signal (Fig. 3). In addition, there was weaker phase-locking of spikes and bursts to the background frequency rhythms present within the input signal (shown as light blue colour in Fig. 3). Notably, the probability of firing a spike or burst at a preferred phase of the dominant frequency band within the input signal was consistently greater than the probability of firing relative to the phase of other rhythms. In particular, the probability of firing an *n*-spike burst at the preferred phase of the dominant rhythm (red colours in Fig. 3) was approximately two to four times greater than the firing probability at a preferred phase of background rhythms (light blue colours in Fig. 3).

In all simulations, there was a shift in phase-locking as a function of burst size *n*. When the input signal contained a peak at 1 Hz, firing of single spikes relative to the dominant slow oscillations was concentrated around a preferred phase of 13°±41° (Fig. 3A). Phase-locking of two-spike bursts and larger bursts advanced by 20° and 30° (preferred phases of 33°±38° and 43°±29°, Fig. 3B and C), respectively, relative to the preferred phase of single spikes. When rhythms of 4 Hz were dominant, locking of single spikes relative to low theta rhythms was around a preferred phase of 11°±30° (Fig. 3D). Two-spike bursts were preferentially fired more advance in phase by 28° (preferred phase of 39°±20°, Fig. 3E) and larger bursts were an additional 15° more advanced (preferred phase of 54°±13°, Fig. 3F). When the input contained a peak at 8 Hz or 12 Hz, single spikes were locked at a preferred phase of −14°±28° of high theta rhythms (Fig. 3G) or −25°±27° of alpha rhythms (Fig. 3J), respectively. Locking of two-spike bursts was advanced by 36° and 39° (preferred phases of 22°±19° and 14°±18°, Fig. 3H and K), respectively, relative to the preferred phase of single spikes. Locking of larger bursts was further advanced by 19° and 23° (preferred phases of 41°±13° and 37°±14°, Fig. 3I and L), respectively, relative to the preferred phase of two-spike bursts.

### 3.4 Bursting neuronal firing is phase-locked to dominant LFP rhythms

**Fig. 4:**
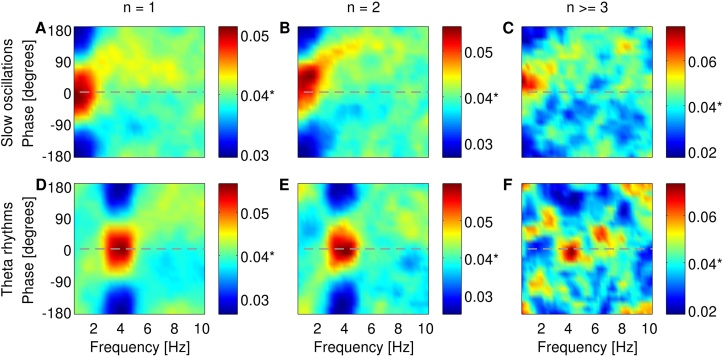
Phase-locking histograms of single spikes (A and D), two-spike bursts (B and E) and larger bursts (C and F) fired by subicular neurons. (A–C) Average across 13 units when slow oscillations were dominant in the LFP signals. (D–F) Average across 11 units when theta rhythms were dominant. The phase of maximal locking of single spikes (*n* = 1) was set to 0° and locking of bursts (*n* = 2 and *n* ≥ 3) was plotted relative to that phase. The colourbar shows the probability of locking to the phase of filtered LFP at overlapping steps of 1 Hz. The asterisk (*) in the colourbar indicates chance probability which is equal to 1/25 or 0.04. (For interpretation of the references to colour in text, the reader is referred to the web version of the article.)

We tested the model predictions by studying how bursting neurons in the rat subiculum fire spikes and bursts in relation to the phase of LFP recorded at the same electrode where bursting activity was recorded. Figs. S3, S4 and 4 show the probability of firing single spikes (*n* = 1), two-spike bursts (*n* = 2) and larger bursts (*n* > =3) at each phase of narrowband filtered LFP. Spikes and bursts were fired at a preferred phase of the dominant rhythm within the LFP signal. This preferred phase varied between individual units as illustrated in the examples in Figs. S3 and S4. The preferred phase of firing single spikes was set to 0° (Fig. 4A and D) and the average phase-locking probabilities of *n*-spike bursts are presented relative to that phase . When slow oscillations were the dominant rhythms in the LFP, the probability of firing an (Fig. 4B–C and 4E–F, respectively)*n*-spike burst at the preferred phase of slow oscillations was 20–80% greater than the chance probability (Fig. 4A–C). Similarly, when theta rhythms were dominant, the probability of firing an *n*-spike burst at the preferred phase of theta rhythms was 20–80% greater than the chance probability (Fig. 4D–F). There was also some phase preference at frequencies outside the dominant band (yellow colours in Fig. 4) but this was substantially weaker than the phase preference at dominant frequencies (red colours in Fig. 4). Moreover, there was a shift in phase preference of bursts (*n* = 2 and *n* > =3) compared to single spikes (*n* = 1) when slow oscillations were dominant (Fig. 4A–C). This shift in phase preference was not observed when *n*-spike bursts were fired during theta-dominant epochs (Fig. 4D–F).

## 4 Discussion

We studied the phase-locking properties of bursting neurons using a pyramidal neuron model as well as *in-vivo* recordings of LFP and spiking activity from the rat subiculum. We simulated different LFP states with physiologically relevant rhythms to determine how phase-locking of bursting activity depends on frequency composition of LFP. The model predicted that *n*-spike bursts lock to dominant oscillations in the input signal regardless of the frequency of these oscillations. In particular, the same phase-locking patterns were noticed in simulated states of dominant slow oscillations, low and high theta rhythms, and also persisted when the input signal contained a power spectral peak at 12 Hz which corresponds to the lower boundary of beta rhythms in rodents or upper boundary of alpha rhythms in primates. This suggests that internal cell mechanisms allow bursting pyramidal neurons to lock their firing to dominant LFP rhythms regardless of their specific frequency.

We observed two prominent rhythms within the LFP recorded from the rat subiculum under urethane anaesthesia. These were slow oscillations which are characteristic of slow-wave sleep or non-REM sleep (Wolansky et al., 2006, Clement et al., 2008) and theta rhythms which are present in the hippocampus during REM sleep (Harris et al., 2002) as well as during exploratory behaviour (O’Keefe and Recce, 1993, Skaggs et al., 1996), although under urethane anaesthesia the theta peak at 4 Hz is lower than the theta peak at 7 Hz during REM sleep (Clement et al., 2008). Since these two rhythms correspond to different cognitive states, we analysed bursting activity during epochs of each dominant rhythm separately.

As predicted by the model, subicular neurons fired single spikes, two-spike bursts and larger bursts which were locked at a preferred phase range of the dominant rhythm within the LFP. The preferred phase range of locking was wider for the subicular neurons than the model. This was possibly a consequence of the lower signal-to-noise in experimental data than the simulated data. Although, some weaker phase preference to background rhythms was also observed, locking to rhythms in the dominant frequency band was at least two times stronger than to any other frequency. These results suggest that the distribution of LFP power modulated the strength of phase-locking of bursting neuronal firing. Modulation of neuronal firing by theta rhythms, which have increased power during behavioural tasks, is a known phenomenon in the hippocampal formation. More specifically, theta phase precession of neuronal firing in the hippocampus has been proposed to be a mechanism to encode spatial position (O’Keefe and Recce, 1993, Skaggs et al., 1996) and a buffer for working memories (Lisman and Idiart, 1995). Theta phase precession has also been reported in the subiculum (Kim et al., 2012). Furthermore, organising neuronal firing by high-power slow oscillations during slow-wave sleep is thought to be important for memory consolidation (Lee and Wilson, 2002, Wolansky et al., 2006, Rasch and Born, 2013).

The model also predicted a gradual shift in phase preference as a function of burst size *n* supporting a burst spike-count code in which single spikes and bursts of different sizes can provide more symbols to encode timing information conveyed by LFP. A similar shift in phase-locking of subicular bursting neurons was observed during epochs when slow oscillations were dominant under anaesthesia providing evidence that this code occurs *in vivo*. We did not observe a similar shift when theta rhythms were dominant but this could be due to the anaesthesia affecting theta rhythms. Firing bursts of spikes can have a number of roles as revealed by studies in various brain systems. Thalamic neurons can fire bursts in response to salient stimuli (Guido and Weyand, 1995, Sherman, 2001, Swadlow and Gusev, 2001). Bursting in the hippocampus improves the reliability of synaptic transmission (Lisman, 1997). Bursts fired by electrosensory cells in the weakly electric fish encode different stimuli to those encoded by tonic spikes (Oswald et al., 2004). Firing bursts with different spike counts also provides a graded signal that allows encoding of different stimuli (Kepecs and Lisman, 2003, Samengo et al., 2013). Theoretical studies suggest burst size can encode the slope (Kepecs et al., 2002) and phase (Samengo and Montemurro, 2010) of input signals. In addition, there is experimental evidence that burst size can encode orientation of visual stimuli in the primary visual cortex of awake monkeys (Martinez-Conde et al., 2002) and intensity of auditory stimuli in grasshopper auditory receptor neurons (Eyherabide et al., 2008, Eyherabide et al., 2009). The outcome of our study expands understanding about the role of bursting in the subiculum.

### 4.1 Conclusions

The model suggests phase-locking of *n*-spike bursts is modulated by the power of the rhythms present in the LFP signal, so that locking to dominant rhythms is stronger than to background rhythms. The analysis of experimental data showed that the output of subicular bursting neurons preferentially locked to the phase of slow oscillations and theta rhythms in two distinct states under urethane anaesthesia. Since phase-locking to dominant rhythms was observed regardless of the frequency of these rhythms, locking appears to be a dynamic property of bursting neurons but not a property of the specific frequency at which the locking occurs. This means that burst firing can potentially lock to the dominant frequencies associated with a variety of behaviours. The outcome of this work needs to be explored further in future studies as the present analyses are based on data from one rat. Although we presented example data from the subiculum, the model is more general so can also be applied to understand the properties of bursting in other cortical and subcortical areas containing pyramidal neurons. Similar phase-locking patterns of bursting neuronal firing might occur in other regions of the brain during both sleep and awake states. Therefore, our results suggest that bursting neurons are likely to play a more significant role in the neural code than previously assumed.

## Footnotes

Appendix A Supplementary data associated with this article can be found, in the online version, at http://dx.doi.org/10.1016/j.biosystems.2015.08.004.
