# Supplementary material for: Phase-locking of bursting neuronal firing to dominant LFP frequency components
Source: Biosystems. 2015 Oct;136:73–9. doi: 10.1016/j.biosystems.2015.08.004 (PMC4669304; doi:10.1016/j.biosystems.2015.08.004)
Supplement: Supplementary file 1 [file mmc1.docx]

**Phase-locking of bursting neuronal firing to dominant LFP frequency components**

Maria Constantinou, Daniel H. Elijah, Daniel Squirrell, John Gigg, Marcelo A. Montemurro

**Supplementary data**

**
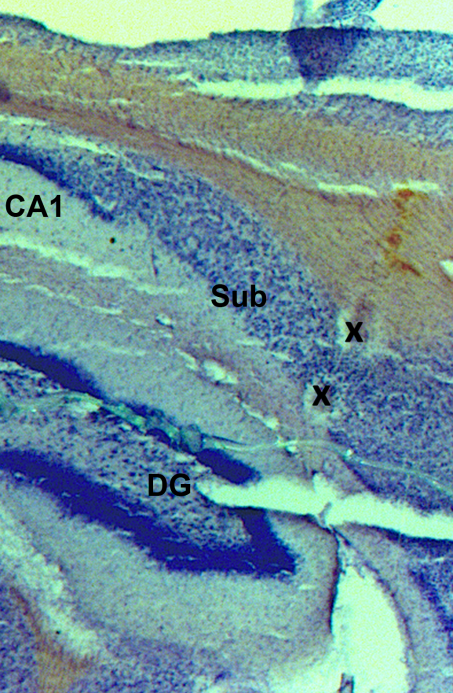
**

**Figure S1.** Nissl-stained brain section showing the position of electrodes in the subiculum. The electrolytic lesions produced at the end of the experiment are marked with an ‘x’ and indicate the position of the first shank of the multi-electrode array. The remaining three shanks are located to the left of these lesions. Sub: subiculum; CA1: hippocampal area CA1; DG: dentate gyrus.


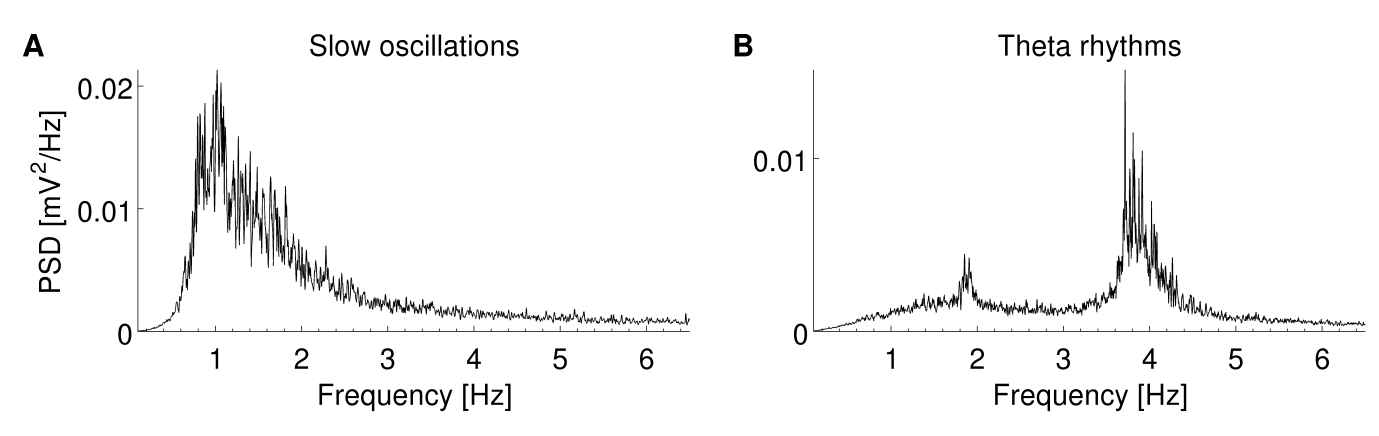
**Figure S2.** Average power-frequency spectra of LFP segments identified as containing dominant slow oscillations (**A**) or theta rhythms (**B**) in the rat subiculum under urethane anaesthesia. **A** The spectrum contained only a peak at slow oscillations indicating there was no remnant theta rhythm. **B** The spectrum contained a large peak at theta rhythms and also a small peak at lower frequencies which was due to coexistence of rhythms in the two bands during theta states under urethane anaesthesia. Spectra plotted using the Welch’s periodogram method with Hamming windows of length at least 130 s and 50% overlap.

**
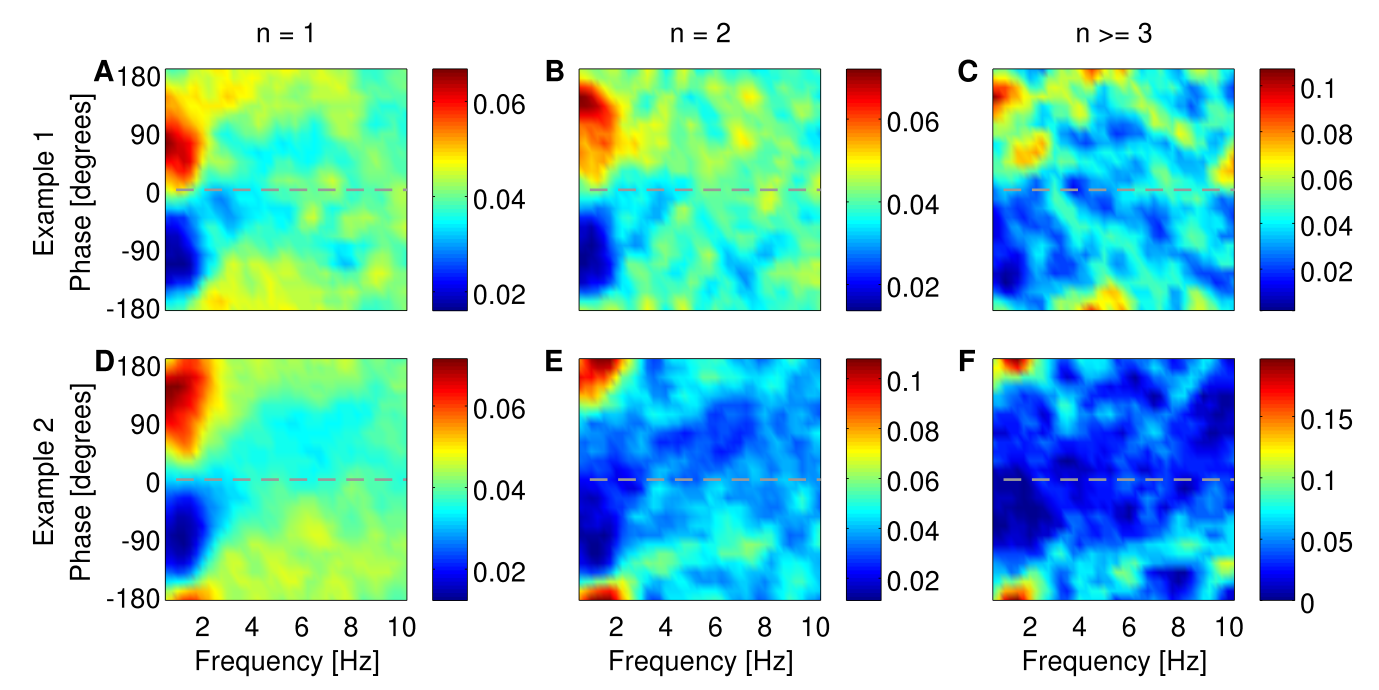
**

**Figure S3.** Phase-locking histograms of single spikes (**A,D**), two-spike bursts (**B,E**) and larger bursts (**C,F**) fired by two different subicular neurons when slow oscillations were dominant in the LFP signals. The preferred phase range of firing differed for each neuron (**A-C** and **D-F**). Phase of 0° corresponds to the peak of a waveform as calculated by the Hilbert transform. The colourbar shows the probability of locking to the phase of filtered LFP at overlapping steps of 1 Hz. Chance probability is equal to 0.04.

**
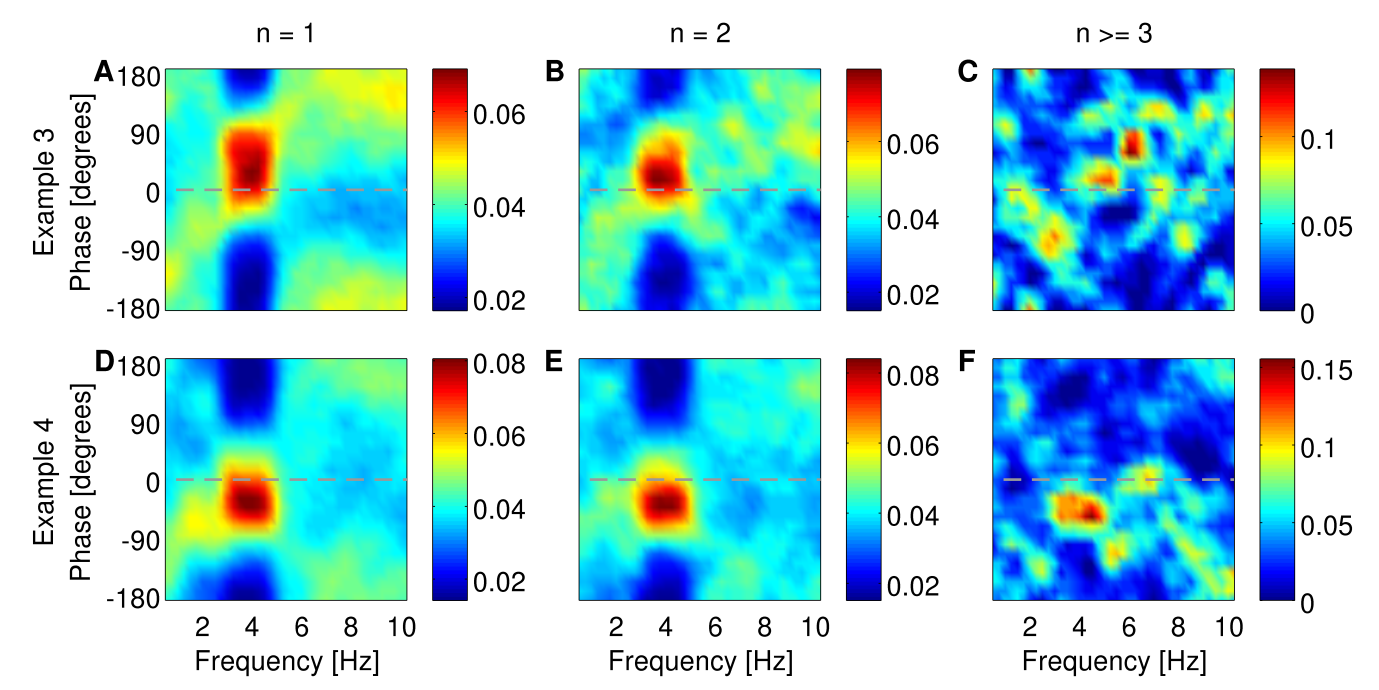
**

**Figure S4.** Phase-locking histograms of single spikes (**A,D**), two-spike bursts (**B,E**) and larger bursts (**C,F**) fired by two different subicular neurons when theta rhythms were dominant in the LFP signals. The preferred phase range of firing differed for each neuron (**A-C** and **D-F**). Phase of 0° corresponds to the peak of a waveform as calculated by the Hilbert transform. The colourbar shows the probability of locking to the phase of filtered LFP at overlapping steps of 1 Hz. Chance probability is equal to 0.04.
